# Use of Intraosseous Vascular Access During Neonatal Resuscitation at a Tertiary Center

**DOI:** 10.3389/fped.2020.571285

**Published:** 2020-09-18

**Authors:** Lukas P. Mileder, Berndt Urlesberger, Bernhard Schwaberger

**Affiliations:** Division of Neonatology, Department of Pediatrics and Adolescent Medicine, Medical University of Graz, Graz, Austria

**Keywords:** neonate, resuscitation, vascular access, intraosseous access, intraosseous emergency infusion

## Abstract

**Introduction:** Emergency vascular access is rarely required during neonatal resuscitation. We aimed to analyze frequency of use, success, and complication rates of intraosseous (IO) vascular access in neonates at a single tertiary neonatal intensive care unit.

**Method:** We performed a questionnaire-based survey among pediatric residents, pediatricians, and neonatologists, asking for the use of IO access in neonates between April 1st, 2015, and April 30th, 2020. We then reviewed electronic patient charts of all identified neonates for demographic data as well as indications and complications of IO puncture.

**Results:** All 41 questionnaires were answered. Nine physicians had attempted IO access 15 times in a total of 12 neonates. Among them were eight term neonates, three preterm neonates, and one former extreme preterm neonate at a post-menstrual age of 42 weeks (m:f = 6:6). The overall success rate was 75%. IO access was attempted primarily during post-natal resuscitation (11/12 neonates, 91.7%) and after unsuccessful peripheral venous puncture (8/12 neonates, 66.7%). It was used to administer adrenaline, fluid and/or blood, and emergency sedation after intubation. Minor short-term complications were reported in three of nine successful IO punctures (33.3%).

**Discussion:** Over the study period of 61 months, IO access was rarely attempted during neonatal resuscitation. Our success rate was lower than reported elsewhere, suggesting that IO puncture may be more challenging in neonates than in older infants and children. No severe short-term complications occurred.

## Introduction

Emergency vascular access and drug administration are rarely needed during neonatal resuscitation. Of 30,839 neonates, 0.12% required chest compressions and/or adrenaline during resuscitation in the delivery room (1). As decreasing gestational age increases the likelihood of delivery room resuscitation, this rate rises up to 2.7% among preterm neonates between 30 and 34 weeks of gestation (2). Among neonates admitted to the neonatal intensive care unit at the Children's Hospital of Philadelphia, frequency of resuscitation was 2.2% (3).

Pediatric advanced life support guidelines recommend inserting an intraosseous (IO) needle “in critically ill children, if attempts at establishing intra-venous (IV) access are unsuccessful after 1 min” (4). However, in comparison to the pediatric population there is scarce evidence related to IO access in neonates. Ellemunter et al. (5) reported about IO access during resuscitation of 27 preterm and term neonates within 5 h after birth. The initial success rate was 100%, three IO needles had to be replaced due to dislocation, and there were not any major complications or long-term side effects in this cohort (5). Glaeser et al. (6) reported 23 instances of IO access in neonates, with successful insertions in 78% of infants below 1 year of age.

Randomized trials for the evaluation of IO access are still missing. Yet, there are several simulation-based studies. In the simulation-based delivery room study by Rajani et al. (7), IO access was achieved in shorter time when compared to umbilical venous catheterization with no differences in technical error rate.

Nevertheless, severe complications have been described following IO access in neonates and infants, including bone fracture, osteomyelitis, compartment syndrome, and limb ischemia requiring amputation (8–10). In the light of the abovementioned ambiguous evidence, we analyzed frequency of use, success, and complication rates of IO access at our institution.

## Materials and Methods

We performed a questionnaire-based survey at our neonatal intensive care unit, followed by a retrospective electronic patient chart review.

### Setting

The Division of Neonatology at the Department of Pediatrics and Adolescent Medicine, Medical University of Graz, Austria, is a tertiary 47-bed neonatal intensive care unit covering 8,000 births a year, 3,500 of them being inborn patients. Post-natal stabilization and neonatal resuscitation is being performed according to current guidelines (11). For administration of emergency drugs during neonatal resuscitation, either umbilical venous catheterization, peripheral venous puncture or IO access is being used, depending on patients' gestational age, birth weight, and the treating physicians' individual decision.

A battery powered IO access device (Arrow EZ-IO, Teleflex Medical Europe Ltd., Ireland) has been in use since April 1st, 2015. Since its introduction, frequent simulation-based practical trainings regarding its utilization have been delivered to physicians and nurses.

### Data Acquisition

For this purpose, we composed an electronic eight-question questionnaire. A pediatric resident and a neonatal nurse, who were both not involved in study design and data analysis, tested the first draft of the questionnaire for clarity of language and content. Finalized questionnaires were sent by e-mail to all pediatric residents, pediatricians, and neonatologists with clinical duties at our institution between April 1st, 2015, and April 30th, 2020. Participation was voluntary. If there was no response after 2 weeks, we contacted colleagues personally.

Based on the answered questionnaires, we identified patients from electronic medical records and retrospectively collected demographic data as well as indications and complications of IO puncture. We defined success with IO access by (i) correct local puncture and (ii) successful administration of medication and/or fluid, based on questionnaire reports and patient chart review.

### Statistical Analysis

We used Microsoft Excel 2015 (Microsoft Corporation, United States of America) for descriptive data analysis. Numerical data are presented as absolute and relative values.

## Results

All 41 forwarded questionnaires (100%) were answered and returned. During the study period of 61 months, nine of the 41 physicians (22.0%) had attempted IO access 15 times in a total of 12 neonates. Six of the 15 IO access attempts (40%) had been undertaken by residents and nine (60%) by fellows and neonatologists, respectively. All punctures were attempted at the proximal tibia. Eight of the 12 patients were term neonates, three were preterm neonates, and one former extreme preterm neonate received IO access at a post-menstrual age of 42 weeks. Ten of the 12 neonates (83.3%) required IO access during post-natal transition in the neonatal resuscitation suite, while the other two patients (16.7%) had IO access established at the neonatal intensive care unit. Demographic patient data and clinical outcomes are summarized in Table 1.

**Table 1:** Demographic data and clinical characteristics of analyzed neonates.

**Patient number**	**Sex**	**Gestational age (weeks)**	**Birth weight (grams)**	**Delivery mode**	**Apgar 1/5/10**	**Day of IO access attempt**	**IO access successful**	**Diagnosis**	**Outcome**
1	f	38 + 2	3500	CS	0/0/0	1	Yes (2nd attempt)	Placenta abruption, prenatal asphyxia	Died day 1
2	m	36 + 6	3000	CS	0/1/1	1	Yes	Perinatal asphyxia with HIE III	Survived to discharge
3	f	41 + 2	4642	CS	0/0/0	1	Yes	Placenta abruption, perinatal asphyxia	Died day 1
4	f	24 + 5	730	VD	n.d.	121	Yes	Severe BPD, PHT, acute hypovolemia due to norovirus infection	Died before discharge
5	m	40 + 6	3400	CS	2/1/0	1	Yes (2nd attempt)	Perinatal asphyxia, long-QT syndrome	Survived to discharge
6	m	40 + 2	4120	VE	0/5/7	1	No (1 attempt)	Shoulder dystocia, perinatal acidosis	Survived to discharge
7	m	36 + 0	2800	CS	0/0/0	1	No (1 attempt)	Umbilical cord tear, perinatal asphyxia	Died day 1
8	f	41 + 3	3550	VD	9/10/10	1	No (1 attempt)	Post-natal asphyxia, right ventricular hypertrophy, pulmonary sclerosis, PHT	Died day 2
9	f	39 + 4	3000	CS	0/0/0	1	Yes	Perinatal asphyxia	Died day 3
10	m	38 + 1	2800	CS	4/4/6	1	Yes (2nd attempt)	Congenital diaphragmatic hernia, omphalocele, scoliosis	Died day 1
11	m	35 + 3	2600	CS	6/3/4	1	Yes	Perinatal acidosis, cerebral infarction	Survived to discharge
12	f	37 + 2	n.d.	CS	5/3/2	1	Yes	Congenital diaphragmatic hernia	Died day 1

*BPD, bronchopulmonary dysplasia; CS, cesarean section; HIE, hypoxic ischemic encephalopathy; IO, intraosseous; n.d., not documented; PHT, pulmonary hypertension; VD, vaginal delivery; VE, vacuum extraction (vacuum-assisted vaginal delivery)*.

Eight of the nine physicians (88.9%) had previously trained IO access using simulation-based methods. IO access could be successfully gained in nine of 12 patients (75.0%). In six of the 12 neonates (50.0%) IO access was successful on the first attempt, while in three further neonates (25.0%) it was successful on the second attempt. In the remaining three patients (25.0%) an alternative vascular access route was used after one unsuccessful attempt.

IO access was attempted during post-natal resuscitation in 11 neonates (91.7%). In eight of the 12 neonates (66.7%) IO access was attempted after unsuccessful peripheral venous puncture and in four patients (33.3%) it was attempted primarily. IO access was used to administer adrenaline (*n* = 5), fluid and/or blood (*n* = 3), and emergency sedation after intubation (*n* = 1). Minor short-term complications (paravasation, local skin reactions and/or local soft tissue infections) were reported in three of nine successful IO punctures (33.3%; Supplementary Figure 1).

## Discussion

In general, IO access was rarely required during the study period, with a mean of 3.0 attempts per year at our institution. The success rate of IO access was lower compared to previous reports with heterogeneous patient cohorts. In 30 children aged between 2 weeks and 9 years, IO access was successfully achieved in every patient with a first-attempt success rate above 80% (12). Among critically ill children with a mean patient age of 5.5 years, IO access could be successfully gained in 94% (13). In severely dehydrated children ranging from 3 months to 2 years of age, all of those assigned to receive IO fluid resuscitation had IO access successfully secured within the first 5 min (14).

The lower rate of success in our cohort—especially on the first attempt—may be explained by neonates' small medullar cavity in comparison to children. A radiographic study in term neonates found mean medullary diameters of 7.7 ± 0.4 mm (anterior-posterior) and 7.4 ± 0.7 mm (lateral) at the proximal tibia (10), emphasizing the need of correct anatomical landmark identification before puncture. In neonates as well as in children, IO puncture should be performed at the antero-medial surface of the tibia one to two centimeters below the tibial tuberosity (10).

Another reason for our lower success rate may be the IO access device itself. Fuchs et al. (15) reported highest success rates with the manual technique using a Butterfly needle (61.1%), followed by hand-twisted EZ-IO needle insertion (43%) and then the semi-automatic needle insertion using the Arrow EZ-IO system (39.7%). However, it has to be noted that our clinical results using the semi-automatic Arrow EZ-IO system were considerably better than the ones from Fuchs et al. (15) in preterm and term stillborns.

Although the majority of physicians had trained IO needle placement prior to its actual clinical use, the overall success rate was only 75% in our study. Hence, contrary to other skills such as bag-valve-mask ventilation (16), neonatal IO access may require more extensive and/or more regular training to achieve competency. A randomized controlled trial found simulation-based multidisciplinary team training at intervals of 6 weeks compared to 6 months to result in significantly higher IO success rates (17). Low-dose high-frequency training has been shown to improve skill retention and may further help improving IO success rates (18). Therefore, we will aim at integrating brief simulation-based IO access training to our weekly interprofessional resuscitation exercises at least once per month.

IO access was primarily attempted during neonatal resuscitation after birth, which is in accordance with other reports (5). It is interesting to note that IO access was mainly attempted after unsuccessful peripheral venous puncture, although the majority of our patients was in critical cardio-circulatory condition and required resuscitation. While peripheral intravenous access during post-natal stabilization of preterm neonates is successful in most cases at first attempt (19), it could only be gained in 17% of infants presenting in cardiac arrest at a mean age of 5 months (20).

Minor short-term complications associated with IO access occurred in 33.3% of cases, but none necessitated specific therapeutic intervention. We cannot report on potential long-term sequelae of IO access. Nevertheless, Claudet et al. (21) found no long-term negative effect on tibial growth after emergency infusion into bone marrow.

In their recent systematic literature review, Scrivens et al. (22) summarized IO access in a total of 64 neonates. While acknowledging that no study so far has directly compared umbilical venous catheterization to IO access in neonates, the authors concluded that IO access “should be available on neonatal units and considered for early use in neonates where other access routes have failed” (22). Accordingly, another literature review recommended IO access to “be considered in situations with difficulty in establishing other access” (23). Although we have to point out the moderate success rate in our study, we can add to this body of evidence supporting the usability of IO access in late preterm and term neonates.

### Limitations and Strengths

We identified patients requiring IO access through a questionnaire-based survey and cannot completely rule out inconsistencies in data reporting. On the other hand, the high questionnaire return rate should allow for a fair representation of the use of IO access at our institution. Furthermore, we verified responses from the survey through patient chart review in order to improve the quality of study data.

## Conclusions

IO access was rarely attempted during neonatal resuscitation. Despite offering regular simulation-based training, our success rate was lower than reported by other groups, suggesting that IO access in neonates may be more challenging to achieve than in older infants and children. While short-term complications were reported in a third of patients, no severe complications occurred.

## Data Availability Statement

The data analyzed in this study is subject to the following licenses/restrictions: research data is confidential. Deidentified participant data will be made available to researchers upon reasonable request to the study authors. Requests to access these datasets should be directed to Lukas P. Mileder, lukas.mileder@medunigraz.at.

## Ethics Statement

Ethical review and approval was not required for the study on human participants in accordance with the local legislation and institutional requirements. Written informed consent from the participants' legal guardian/next of kin was not required to participate in this study in accordance with the national legislation and the institutional requirements.

## Author Contributions

LM conceptualized the study, collected the patient data, analyzed the data, drafted the initial manuscript, and revised the manuscript. BU coordinated and supervised the data collection, analyzed the data, critically reviewed, and revised the manuscript for important intellectual content. BS conceptualized the study, collected the patient data, analyzed the data, and revised the manuscript for important intellectual content. All authors approved the final manuscript as submitted and agree to be accountable for all aspects of the work.

## Conflict of Interest

The authors declare that the research was conducted in the absence of any commercial or financial relationships that could be construed as a potential conflict of interest.

## References

[1] Perlman JM Risser R. Cardiopulmonary resuscitation in the delivery room. Associated clinical events. Arch Pediatr Adolesc Med. (1995) 149:20–5. 10.1001/archpedi.1995.02170130022005 7827654

[2] Bajaj M Natarajan G Shankaran S Wyckoff M Laptook AR Bell EF. Delivery room resuscitation and short-term outcomes in moderately preterm infants. J Pediatr. (2018) 195:33–8.e2. 10.1016/j.jpeds.2017.11.039 29306493 PMC5869086

[3] Foglia EE Langeveld R Heimall L Deveney A Ades A Jensen EA. Incidence, characteristics, and survival following cardiopulmonary resuscitation in the quaternary neonatal intensive care unit. Resuscitation. (2017) 110:32–6. 10.1016/j.resuscitation.2016.10.012 27984153 PMC5193233

[4] Maconochie IK Bingham R Eich C López-Herce J Rodríguez-Núñez A Rajka T. European Resuscitation Council guidelines for resuscitation 2015: section 6. Paediatric life support. Resuscitation. (2015) 95:223–48. 10.1016/j.resuscitation.2015.07.028 26477414

[5] Ellemunter H Simma B Trawöger R Maurer H. Intraosseous lines in preterm and full term neonates. Arch Dis Child Fetal Neonatal Ed. (1999) 80:F74–5. 10.1136/fn.80.1.F74 10325819 PMC1720869

[6] Glaeser PW Hellmich TR Szewczuga D Losek JD Smith DS. Five-year experience in prehospital intraosseous infusions in children and adults. Ann Emerg Med. (1993) 22:1119–24. 10.1016/S0196-0644(05)80975-8 8517560

[7] Rajani AK Chitkara R Oehlert J Halamek LP. Comparison of umbilical venous and intraosseous access during simulated neonatal resuscitation. Pediatrics. (2011) 128:e954–8. 10.1542/peds.2011-0657 21930542

[8] La Fleche FR Slepin MJ Vargas J Milzman DP. Iatrogenic bilateral tibial fractures after intraosseous infusion attempts in a 3-month-old infant. Ann Emerg Med. (1989) 18:1099–101. 10.1016/S0196-0644(89)80937-0 2802286

[9] Stoll E Golej J Burda G Hermon M Boigner H Trittenwein G. Osteomyelitis at the injection site of adrenalin through an intraosseous needle in a 3-month-old infant. Resuscitation. (2002) 53:315–8. 10.1016/S0300-9572(02)00039-4 12062848

[10] Suominen PK Nurmi E Lauerma K. Intraosseous access in neonates and infants: risk of severe complications–a case report. Acta Anaesthesiol Scand. (2015) 59:1389–93. 10.1111/aas.12602 26300243

[11] Wyllie J Bruinenberg J Roehr CC Rüdiger M Trevisanuto D Urlesberger B. European Resuscitation Council guidelines for resuscitation 2015: section 7. Resuscitation and support of transition of babies at birth. Resuscitation. (2015) 95:249–63. 10.1016/j.resuscitation.2015.07.029 26477415

[12] Claudet I Fries F Bloom MC Lelong-Tissier MC. Retrospective study of 32 cases of intraosseous perfusion. Arch Pediatr. (1999) 6:516–9. 10.1016/S0929-693X(99)80557-0 10370806

[13] Horton MA Beamer C. Powered intraosseous insertion provides safe and effective vascular access for pediatric emergency patients. Pediatr Emerg Care. (2008) 24:347–50. 10.1097/PEC.0b013e318177a6fe 18562874

[14] Banerjee S Singhi SC Singh S Singh M. The intraosseous route is a suitable alternative to intravenous route for fluid resuscitation in severely dehydrated children. Indian Pediatr. (1994) 31:1511–20. 7875811

[15] Fuchs Z Scaal M Haverkamp H Koerber F Persigehl T Eifinger F. Anatomical investigations on intraosseous access in stillborns–comparison of different devices and techniques. Resuscitation. (2018) 127:79–82. 10.1016/j.resuscitation.2018.04.003 29627398

[16] van Vonderen JJ Witlox RS Kraaij S te Pas AB. Two-minute training for improving neonatal bag and mask ventilation. PLoS ONE. (2014) 9:e109049. 10.1371/journal.pone.0109049 25279800 PMC4184825

[17] Ghazali DA Fournier E Breque C Ragot SP Oriot D. Immersive simulation training at 6-week intervals for 1 year and multidisciplinary team performance scores: a randomized controlled trial of simulation training for life-threatening pediatric emergencies. Emergencias. (2019) 31:391–8. 31777210

[18] Sutton RM Niles D Meaney PA Aplenc R French B Abella BS. Low-dose, high-frequency CPR training improves skill retention of in-hospital pediatric providers. Pediatrics. (2011) 128:e145–51. 10.1542/peds.2010-2105 21646262 PMC3387915

[19] Baik-Schneditz N Pichler G Schwaberger B Mileder L Avian A Urlesberger B. Peripheral intravenous access in preterm neonates during postnatal stabilization: feasibility and safety. Front Pediatr. (2017) 5:171. 10.3389/fped.2017.00171 28848726 PMC5554121

[20] Brunette DD Fischer R. Intravascular access in pediatric cardiac arrest. Am J Emerg Med. (1988) 6:577–9. 10.1016/0735-6757(88)90094-0 3178949

[21] Claudet I Baunin C Laporte-Turpin E Marcoux MO Grouteau E Cahuzac JP. Long-term effects on tibial growth after intraosseous infusion: a prospective, radiographic analysis. Pediatr Emerg Care. (2003) 19:397–401. 10.1097/01.pec.0000101580.65509.5e 14676488

[22] Scrivens A Reynolds PR Emery FE Roberts CT Polglase GR Hooper SB. Use of intraosseous needles in neonates: a systematic review. Neonatology. (2019) 116:305–14. 10.1159/000502212 31658465

[23] Wagner M Olischar M O'Reilly M Goeral K Berger A Cheung PY. Review of routes to administer medication during prolonged neonatal resuscitation. Pediatr Crit Care Med. (2018) 19:332–8. 10.1097/PCC.0000000000001493 29406382

