# Supplementary material for: Use of Intraosseous Vascular Access During Neonatal Resuscitation at a Tertiary Center
Source: Front Pediatr. 2020 Sep 18;8:571285. doi: 10.3389/fped.2020.571285 (PMC7530188; doi:10.3389/fped.2020.571285)

Supplementary Figure 1. Local skin reaction following successful intraosseous blood administration, without any negative long-term consequences. Note the puncture site at the anterior aspect of the tibia rather than at the antero-medial surface of the tibia.

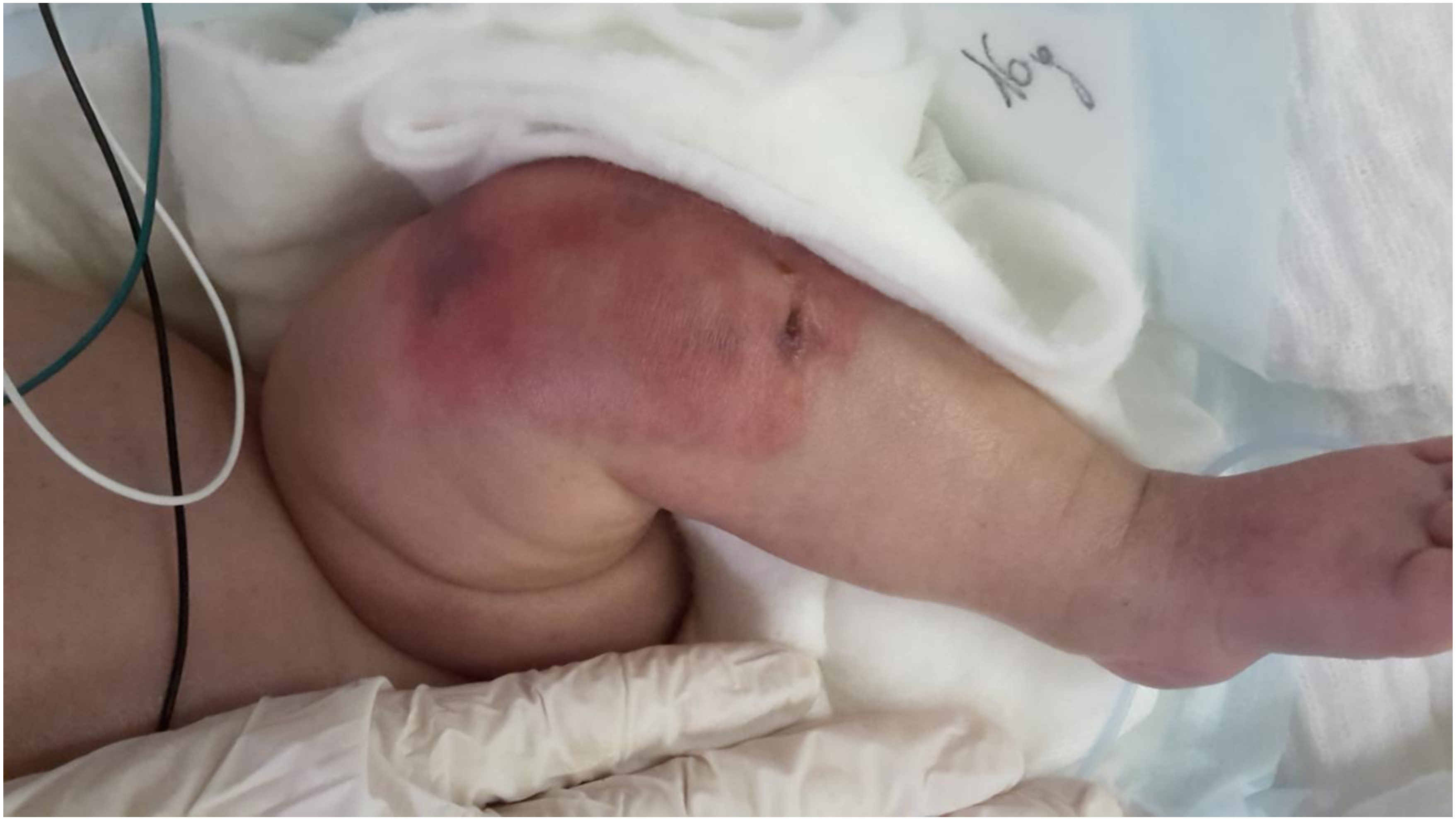

Supplement: Supplementary file 1 [file Image_1.pdf]
